# Evaluating the feasibility and preliminary effects of a group-based telerehabilitation program in individuals with persistent dyspnea after COVID-19 (TEPCO): a pilot randomized controlled study

**DOI:** 10.1186/s40814-026-01876-w

**Published:** 2026-07-07

**Authors:** Marie-Claude Lehoux, Mariève Houle, Didier Saey, Charles Tétreau, Alice Lefebvre, Eva Mathieu, Christine Drapeau

**Affiliations:** 1 Centre intégré universitaire de santé et de services sociaux de la Mauricie-et-du-Centre-du-Québec (CIUSSS MCQ), Infrastructure de recherche en prévention et promotion de la santé (IRPPS), Trois-Rivières, QC Canada; 2 Départment d’anatomie, Université du Québec à Trois-Rivières, Trois-Rivières, QC Canada; 3 Centre de Recherche, Institut Universitaire de Cardiologie et de Pneumologie de Québec, Université Laval, Québec, QC Canada; 4 Département des sciences de l’activité physique, Université du Québec à Trois-Rivières, Trois-Rivières, QC Canada; 5 Département de Pneumologie, Centre Hospitalier Affilié Universitaire de Trois-Rivières, Trois-Rivières, QC Canada

## Abstract

**Background:**

Many COVID-19 survivors have been shown to experience at least one persistent symptom or deficit following infection, impacting their functioning and quality of life. However, knowledge about the appropriate care and rehabilitation needs of individuals with Long COVID remains fragmented. More research is needed to understand the impact of rehabilitation or telerehabilitation programs on Long COVID symptoms, quality of life, and work ability.

This is a pilot randomized controlled trial to evaluate the feasibility of the study procedures and to compare the preliminary effects of a rehabilitation program delivered remotely (experimental group) or in-person (control group) for an adult (≥ 18 years) with confirmed COVID-19 and persistent dyspnea at least eight weeks post-infection.

**Methods:**

Participants were randomly assigned to either the telerehabilitation or in-person group using a computer-generated allocation sequence. An 8-week rehabilitation program, including cardiovascular, muscular, balance training, and respiratory exercises, was provided to both groups. Outcomes assessed included feasibility (recruitment rate, dropout rate, adherence rate, occurrence of adverse events, and patient satisfaction). Secondary outcomes comprised preliminary effect measures including exercise capacity, functional capacity, functional independence in daily life, quality of life, and clinical frailty.

**Results:**

Twenty-one participants aged between 29 and 75 (median age 52) were recruited to the study. Thirty-nine (39) eligible individuals were approached, and 22 participants consented to participate in the study (56.4% consent rate). One participant withdrew before the start of the program due to scheduling issues, resulting in 21 participants included in the analysis. Eight participants in the in-person group and ten participants in the telerehabilitation group attended at least 75% of the sessions. No adverse events related to the intervention were reported during the study period. Participants’ satisfaction levels were high, with 90% strongly agreeing to the quality of services received in the telerehabilitation group. For the exercise capacity (pedaling time), there was a Group*Time interaction (*F*_(1, 16)_ = 6.1, ηp^2^ = .28) and a main effect of time (*F*_(1, 16)_ = 20.9, ηp^2^ = .57). A main effect of time was also detected for the One-Minute Sit-to-Stand Test (*F*(1, 16) = 8.1, ηp^2^ = .34) in both groups.

**Conclusions:**

This pilot randomized controlled study demonstrated the feasibility and safety of delivering a supervised group-based rehabilitation intervention remotely compared with in-person delivery to individuals with Long COVID characterized by persistent dyspnea eight weeks post-infection. These findings apply to a relatively young, non-hospitalized population and may not be generalizable to all Long COVID phenotypes. The protocol proved feasible in our clinic, showing favorable recruitment, dropout, and adherence rates, as well as a low occurrence of adverse events and high satisfaction. Preliminary observations on clinical outcomes suggest potential benefits of telerehabilitation compared to in-person delivery; these findings are exploratory and intended to inform future research rather than provide definitive conclusions. Further studies, including larger multicenter RCTs across Québec regions, are needed to evaluate the effects, safety and generalizability of supervised group-based telerehabilitation interventions for individuals with Long COVID.

**Trial registration:**

ClinicalTrial.gov, NCT06952127. Registered on 28 April 2025—retrospectively registered. https://clinicaltrials.gov/study/NCT06952127

## Key message regarding feasibility

- Which uncertainties existed regarding the feasibility?

There were uncertainties regarding the feasibility of implementing a group-based telerehabilitation program for participants with Long COVID in our region. Additionally, there was uncertainty about identifying practical outcome measures capable of detecting meaningful changes for use as primary outcomes, given the lack of validated instruments specifically designed for Long COVID. This gap could limit the selection of appropriate primary outcomes and reduce the sensitivity to detect meaningful changes in future trials. Concerns also existed about the feasibility of a main trial, particularly in terms of recruitment and retention rates relative to sample size requirements.

- What are the key feasibility findings?

Results show that group-based telerehabilitation for participants with Long COVID was feasible, with excellent patient satisfaction, low attrition, and high adherence. Safety was assessed by monitoring adverse events throughout the intervention period in both groups, and none were reported. Recruitment could likely have been improved with a dedicated kinesiologist working full-time with people with chronic health problems and through digital recruitment strategies.

- What are the implications of the feasibility findings for the design of the main study?

This study demonstrates that the proposed methodology for a full randomized control trial study of patients with Long COVID is acceptable and feasible. Refinement in recruitment strategies is necessary, along with selecting an appropriate primary outcome measure to effectively evaluate change.

## Introduction

Over the past five years, knowledge of Severe Acute Respiratory Syndrome Coronavirus 2 (SARS-CoV-2) has evolved considerably. The growing body of scientific literature has improved understanding of the clinical features and management of COVID-19, encompassing both acute illness and long-term sequelae. For instance, it has been demonstrated in several systematic reviews and meta-analyses that many COVID-19 survivors experienced at least one persistent symptom or one deficit following infection [1–3] affecting their functioning and quality of life [4]. In this article, we use the term Long COVID and define it as symptoms persisting for at least eight weeks after the acute episode of COVID-19 [5].

However, information regarding the appropriate post-infectious care and rehabilitation needs for individuals with Long COVID remain fragmented [6, 7]. The U.S. Centers for Disease Control and Prevention (CDC) recommends prioritizing rehabilitation for patients recovering from a SARS-CoV-2 infection; however, the available evidence mainly concerns individuals with severe acute COVID-19 [8]. While rehabilitation is generally thought to improve physical condition and respiratory function in hospitalized COVID-19 patients [9], potential caveats remain for certain post-infectious symptoms, which may require adapted strategies. Nevertheless, there remains a significant gap in understanding the criteria for selecting patients who require rehabilitative interventions, as current evidence mainly comes from studies on in-patients early after COVID-19 [10]. There is also insufficient information on the best strategy for rehabilitation even if home-based programs and telerehabilitation applications are commonly used in the management of persisting symptoms after COVID-19 [11]. The evidence regarding the effects and benefits of rehabilitation and telerehabilitation programs on COVID-19 symptoms, quality of life, and work ability requires further investigation [10, 12–14].

At the time of this research project in 2022, the city of Trois-Rivières, located in the province of Québec (Canada), did not have a specialized clinic for the treatment of Long COVID. For the most part, patients with Long COVID were therefore referred by a primary care provider to specialists in pneumology, neurology, and/or cardiology. To address this gap, a pilot rehabilitation program was developed in collaboration with an academic kinesiology clinic, combining in-person and telerehabilitation modalities.

Telerehabilitation has been shown to improve access to healthcare and service delivery for patients who are geographically or socially isolated, those who work or study full-time, and those for whom travel is challenging due to the severity of their illness or comorbidities [15]. The COVID-19 pandemic highlighted the importance of telerehabilitation as a practical tool for ensuring continuity of pulmonary rehabilitation care for patients who were unable to receive it in-person due to the pandemic or for geographical reasons [16]. Additional advantages include minimizing the risk of reinfection compared with in-person sessions and reducing the physical burden associated with travel to rehabilitation facilities [17]. Recent systematic reviews and randomized trials have reported that telerehabilitation can improve physical function, dyspnea, and quality of life in individuals with Long COVID, with safety comparable to in-person care [18, 19]. However, evidence comparing telerehabilitation and traditional in-person rehabilitation remains limited and heterogeneous, highlighting the need for studies that explore feasibility and preliminary effects of these delivery models in real-world settings.

The overall strategy for this pilot project was therefore to develop and deliver a telerehabilitation program for patients with Long COVID, to improve their functional abilities and quality of life. However, as telerehabilitation is not currently widely used within the facility, it was necessary to evaluate the feasibility of this approach on a smaller scale and observe the effects before being able to implement it on a larger scale.

The purpose of this pilot randomized controlled trial (RCT) was to evaluate the feasibility of the study procedures and to compare the preliminary effects of a rehabilitation delivered remotely (experimental group) or in-person (control group) for improving exercise capacity, functional capacity and independence, clinical frailty, and quality of life in individuals with persistent dyspnea at least eight weeks after COVID-19 infection.

We hypothesized that the study procedures would be feasible and that both groups would have similar results for the preliminary effects outcomes.

## Methods

### Design and setting

The study was a parallel, two-arm pilot randomized controlled trial adhering to the Consolidated Standards of Reporting Trials extension for randomized pilot and feasibility trials [20]. The study took place in the Trois-Rivières area. At the time of the study, no structured rehabilitation program was available within the hospital, so a partnership was established with the kinesiology clinic at *Université du Québec à Trois-Rivières* (UQTR) to deliver the intervention. The clinical evaluations took place either at the hospital’s pulmonology clinic, while the training program was delivered either virtually to participants at home remotely or in-person at the University of Trois-Rivières' kinesiology clinic, depending on their assigned/randomized group. This study was approved by the Institutional Review Board (IRB) (number 2023–667) and all participants provided written informed consent.

### Participants

The participants were adult patients (≥ 18 years) who had confirmed SARS-CoV-2 infection defined by positive polymerase chain reaction (PCR) testing and/or antigen test results and were referred to the pneumology clinic for a modified British Medical Research Council (mMRC) dyspnea score [21, 22] of 2–3 after a minimum of eight weeks from infection. Inclusion criteria were: achieving a VO₂max of less than 80% of the predicted value in a cardiovascular exercise test on an cycle ergometer (stationary bike) performed clinically and as part of the routine assessment of these patients; receiving a medical prescription for rehabilitation following initial assessment by a pulmonologist; being able to walk independently; providing informed consent; having a sufficient internet connection (i.e., a minimum download speed of 4 Mbps and an upload speed of 4 Mbps); and being in possession of a computer or tablet equipped with a camera enabling videoconferencing. Exclusion criteria were: requiring in-patient rehabilitation at discharge; evidence of medical instability (i.e., moderate or severe cardiac disease, ischemic or severe hemorrhagic stroke, or neurodegenerative disease, unstable fracture); history of severe cognitive or mental impairment; and/or already enrolled in another rehabilitation program.

### Recruitment and randomization

Participants meeting the inclusion criteria were identified by the pulmonology team and invited to participate in the research project by a research nurse between April 7, 2023, and November 27, 2023. Twenty-four participants were sought by the research team to obtain a minimum of 20 participants considering a 5% drop-out rate. Finally, 21 patients were recruited during the targeted period. Following informed consent, eligible participants were randomized using a 1:1 allocation ratio to the experimental group (telerehabilitation) or control group (in-person). Simple randomization using the RANDBETWEEN function of Microsoft Excel was implemented by a research professional not involved in clinical assessments. Allocation concealment was ensured by keeping the randomization list inaccessible to the clinical team; group assignments were revealed only after baseline evaluation and consent. Neither participants nor assessors were blinded to group allocation due to the nature of the intervention.

### Procedures

Both groups received a rehabilitation program including cardiovascular, muscular, balance training, and respiratory exercises as proposed by the *Institut Universitaire de Cardiologie et de Pneumologie de Québec* (IUCPQ) [23]. It was presented to participants in groups of five or six in a “remote” (experimental group) or “in-person” model. The sessions were therefore comparable between the two groups. Both groups were also supervised by a kinesiology student under the supervision of a certified kinesiologist. For the control group, sessions were held at the university kinesiology clinic, whereas for the experimental group, sessions were held virtually by videoconferencing with a computer or a tablet equipped with a camera (Teams, Microsoft, Redmond, WA, USA).

Rehabilitation was carried out three times a week for eight weeks. The average session lasted 60 min and was scheduled at a convenient time for the group of participants and the kinesiology student. In the event of discomfort, training was stopped immediately for the affected participant. The number of sessions and their average duration were the same for both groups.

Rehabilitation was adapted for patients with Long COVID according to recommendations issued by the Canadian Thoracic Society [24]. Strategies included lower intensities for aerobic exercise, conservative progression, and careful symptom monitoring, along with gradual introduction of strengthening exercises using resistance bands and bodyweight. To maintain motivation and avoid monotony, a variety of aerobic and strength exercises were used across sessions. Exercise intensity was mainly controlled using the rate of perceived exertion (RPE) assessed with the modified Borg Scale (0–10) developed at IUCPQ by Jean Jobin [25], adapted from Borg G.A.V. [26], with a target of 3–4 out of 10, based on dyspnea, muscle fatigue, and perspiration during the sessions.

Each session started with a 5-min warm-up including easy global exercises and dynamic stretching. Next, five resistance training exercises targeting upper and lower muscle groups were performed, with each exercise consisting of 2 to 3 sets of 5 to 20 repetitions. There was a minimum of a 30-s rest between each set. A cooldown was achieved through breathing and stretching exercises kept for two sets of 20 s. Table 1 shows the exercise intervention summarized as per the Consensus in Exercise Reporting Template (CERT).

**Table 1:** Exercise intervention summarized as per the Consensus in Exercise Reporting Template (CERT)

Item	Description
Name	Group-based rehabilitation in-person AND group-based telerehabilitation
Why: Rationale	To evaluate the feasibility of a group-based telerehabilitation intervention for patients with persistent COVID-19 symptoms
What: Materials	• Hardware: Wi-Fi, webcam on computer/tablet/telephone • Software: Video-conferencing platform (Microsoft Teams) • Equipment: Resistance exercise bands supplied to participants
What: Procedures	• Exercise prescription plan by a certified kinesiologist based on the proposed rehabilitation program of the *Institut Universitaire de Cardiologie et de Pneumologie de Québec* [23] • Pre-procedure outcome measures completed (T0) Three exercise sessions per week over 8 weeks • Low impact, low intensity (maximal rate of perceived exertion (RPE) of 3–4 out of 10) • Full-body resistance training, including functional exercises combined with cardiovascular training, balance, and breathing exercises • Procedure outcome measure at 8 weeks post-completion (T1)
Who: Provider	Kinesiology student under the supervision of a certified kinesiologist
How: Delivery	2 × in-person sessions: Completion of outcome measures 3 × group exercise sessions per week over 8 weeks-in-person or telerehabilitation
Where: Location	Participants in their homes or at the kinesiology clinic and a kinesiology student in the kinesiology clinic-based
When, how much: Dosage	• Frequency: three times per week • Low intensity as per the rate RPE scale of a maximum of 3–4 out of 10 according to dyspnea, muscle fatigue, and amount of perspiration during the sessions • Warm-up: easy global exercises and dynamic stretching for 5 min • Resistance training volume: 2–3 sets of 5 to 20 reps per exercise for 5 exercises • Aerobic exercise: between 2 sets of 90 s (first week) to 2 sets of 5 min (week 8) • Session duration: 60 min
Tailoring: What and how	• Starting point: depending on the type of exercise, body weight or resistance band used (light or medium) determined by the kinesiology student • Progression: number of repetitions and/or series increased each week according to participants' tolerance • Advice from the kinesiology student offering easier or more difficult alternatives to maintain an RPE of maximum 3–4 out of 10
How well: Planned	• Kinesiology student to keep notes on each session and their own diary • Participants are to keep notes about their experience and feelings about the exercise program
How well: Actual	Evaluation of quantitative findings to inform feasibility, safety, satisfaction, occurrence of adverse events, and preliminary effects of intervention

### Measurements

Participants were assessed just before the start of the rehabilitation program. Final assessments were conducted 4 to 6 weeks after the end of the rehabilitation program. This timing allowed for scheduling flexibility and accommodated practical constraints related to clinic and participant availability, while also enabling evaluation of whether improvements were maintained beyond the immediate post-intervention period. All assessments were performed at the Trois-Rivières hospital’s outpatient pulmonology clinic by qualified personnel such as respiratory therapists, nurses, and pulmonologists. The primary outcome was feasibility, including session adherence, patients’ satisfaction, and adverse events. Secondary outcomes comprised preliminary effect outcomes, including exercise capacity, functional capacity, functional independence in daily life, quality of life, and clinical frailty.

### Primary outcome measures

The primary goal of the project was to evaluate the feasibility of the study procedures, which included an in-person or remote rehabilitation program for patients with Long COVID. Feasibility was assessed based on recruitment, attrition, protocol adherence, and adverse events in both groups throughout the intervention period, without formal a priori thresholds, as this pilot study aimed to evaluate feasibility descriptively and inform future trials. Adverse events were defined as any health issues reported during the study. The kinesiology student made sure to record attendance at each rehabilitation session and reasons for participants’ absences in a file. Participants were also asked to complete a satisfaction questionnaire at the end of the program.

The Customer Satisfaction Questionnaire (CSQ-8) [27] was completed by the participants in the control group. The CSQ-8 is a validated measure of general satisfaction with health services, comprising eight statements rated on a four-point Likert scale*.* The experimental group was asked to complete the Telemedicine Patient Satisfaction Questionnaire [28], which assesses patient satisfaction with telemedicine services. This questionnaire has 15 items with no categories, rated on a 5-point Likert scale ranging from 1 (strongly disagree) to 5 (strongly agree). Frequently used in the literature [29], its content and construct have been validated for internal consistency and reliability [30]. Patient satisfaction was assessed using the validated French versions of the CSQ-8 [31, 32], validated in a Canadian French-speaking population, and the Telemedicine Patient Satisfaction Questionnaire [33]. Both instruments have demonstrated good psychometric properties for assessing patient satisfaction in healthcare and telemedicine contexts, respectively.

Adverse events defined as serious or non-serious were recorded in a logbook by the participants during training and/or by the professional during follow-up. A serious adverse event was defined as an event leading to hospitalization, while a non-serious event included musculoskeletal injuries and symptom exacerbations. Symptom exacerbations were defined as a worsening of pre-existing symptoms (e.g., fatigue, dyspnea, pain) following exercise. These were self-reported by participants as free-text notes in a logbook either immediately after training sessions or at any time between sessions, and subsequently recorded by the professional during scheduled follow-up calls. To monitor for adverse events and provide support, each participant received four scheduled phone calls from the research nurse, had access to the team by phone at any time, and was seen by the kinesiologist every 2 days (three training sessions per week).

### Secondary outcome measures

The preliminary effects were verified on clinical outcomes such as exercise capacity, functional ability and independence, clinical frailty, and quality of life. These measures were collected during the clinical assessment before the start of the rehabilitation program (baseline) and immediately after completion of the program for all participants.

### Exercise capacity

Exercise capacity was determined by endurance time performed on a cycle ergometer at constant load (i.e., 60% of maximum load). This load was determined during a progressive-load maximum cardiovascular exercise test on a cycle ergometer carried out at the start of the program. After 3-min rest, the patient cycled at constant speed (60 RPM) and constant load (60% of the patient’s maximum load) until voluntary exhaustion or the patient’s inability to keep up with the pedaling cadence. The professional supervising the exercise test provided continuous verbal encouragement to the individual. Heart rate, blood pressure, saturation, and symptoms of dyspnea and leg fatigue were assessed at rest, every 2 min, at the end of effort, and 5 min after stopping the test. Pedaling time was considered the main test variable.

### Functional capacity and independence

Patients’ functional capacity and independence were assessed using the 6-Min Walk Test (6MWT), the One-Minute Sit-to-Stand Test (1STST), and the Short Physical Performance Battery test (SPPB) [34]. Participation and autonomy in activities of daily living were assessed by the Barthel Index [33].

The 6MWT is a self-paced test of walking capacity. Patients are instructed to walk as far as possible in 6 min along a flat corridor according to the guidelines of the European Respiratory Society (ERS)/American Thoracic Society (ATS) [35]. The test is performed in a 30-m indoor corridor and scored by measuring the total distance walked. The 6MWT is a valid and reasonably reliable measure of physical endurance [36].

The 1STST was performed according to the protocol described by Crook and collaborators [37]. Participants were seated upright with knees and hips flexed at 90°, feet flat on the floor and hip-width apart, and arms crossed across the chest. They were instructed to perform repetitions of standing upright and then sitting down in the same position at a self-paced speed for 1 min. The number of completed sit-to-stand repetitions was recorded. The 1STST is a valid, reliable, and practical tool for measuring exercise capacity in clinical populations [38].

The SPPB assesses physical performance through balance, strength, and gait measurements. It consists of a set of three tests: standing static balance in three positions; lower limb strength and power through getting up and sitting on a chair stands; and walking speed at normal pace [39]. Scores range from 0 to 12, with higher scores indicating better physical function. The validity and reliability of the SPPB for assessing lower-extremity physical function and mobility have been proven in a French-speaking population in Québec [40].

The Barthel Index [35] is a measure of independence in activities of daily living (ADL) across 10 basic domains: bowel, bladder, grooming, toilet use, feeding, transfers, walking, dressing, climbing stairs, and bathing. Each item is scored from 0 to 2 (independent), and total score ranges from 0 to 100, with higher scores indicating greater independence. The Barthel Index has demonstrated adequate reliability and validity [41].

### Clinical frailty

The Clinical Frailty Scale (CFS) is a judgment-based frailty tool that evaluates domains such as comorbidity, function, and cognition to generate a frailty score ranging from 1 (very fit) to 9 (terminally ill) with lower scores indicating less frailty [42]. The CFS is a valid, reliable, and easy-to-use tool that allows healthcare providers to assign a score based only on a standard clinical interview [43]. Although originally validated in geriatric populations, recent studies have supported its use in post-COVID-19 rehabilitation settings; however, it has not been specifically validated for all individuals with Long COVID [44, 45].

### Quality of life

Quality of life was measured using the St. George’s Respiratory Questionnaire (SGRQ), a 50-item instrument designed for individuals with respiratory conditions. It comprises three components—Symptoms, Activity, and Impacts—with scores calculated for each domain and a total score summarizing all responses [46]. Scores range from 0 to 100, where 0 indicates no impairment and higher scores reflect greater limitations. For this study, the French-Canadian version of the SGRQ was used, which has demonstrated excellent test–retest reliability and internal consistency. Although the SGRQ has not been formally validated in Long COVID populations and primarily focuses on respiratory symptoms, it was chosen for its relevance to dyspnea and its strong psychometric properties in respiratory conditions [47].

### Sample size

Following established guidelines that recommend a minimum of 20 participants for feasibility studies [48, 49], we included 21 adults experiencing Long COVID. This sample size was considered sufficient to estimate key feasibility parameters, including recruitment, adherence, attrition, and occurrence of adverse events, and to inform the design and sample size calculation of a future definitive trial.

### Statistical analysis

#### Feasibility components

Feasibility outcomes, such as recruitment rate, attrition, adherence to protocol, and adverse events, are presented using descriptive statistics (e.g., N; %) or qualitative data, as appropriate.

#### Pilot components

The normality of data distribution was assessed using the Kolmogorov–Smirnov test and visual inspection of the corresponding histograms for each dependent variable. Baseline sociodemographic data and characteristics were summarized using descriptive statistics, including mean and standard deviation for continuous variables.

Baseline comparisons between groups for sociodemographic data and baseline characteristics were conducted using two-tailed independent t-tests or Mann–Whitney tests for continuous variables, while chi-square tests were used for categorical variables. As this study is a pilot study with a small sample size, Cohen’s *d* effect size and 95% confidence interval (CI) were reported for *t*-tests, *z*-score and effect size (*r*) were reported for Mann–Whitney tests, and phi effect size with corresponding 95% confidence intervals was calculated for chi-square tests where applicable. Between-group comparisons over time for pedaling time, 6MWT, and 1STST were conducted using a mixed-design ANOVA. Effect size (ηp^2^) and 95% CI were reported for the main effect of time and group. Between-group comparisons for SPPB, Barthel Index, and CFS were unnecessary since the scores remained the same across groups over time. For the quality of life questionnaire, missing data on the baseline prevented us from making a comparison between the groups or the difference between before and after the rehabilitation program. Statistical analyses were performed using SPSS version 29 for Windows (IBM, Armonk, NY, USA).

## Results

### Characteristics of participants

Participants' details are presented in Table 2. Nine male and twelve female participants aged between 29 and 75 (median age 52) were recruited to the study. Twenty participants (95%) reported having experienced moderate COVID-19, and one reported having a severe case (severity was self-reported based on symptom intensity and functional impact, not hospitalization status). Of the 21 participants included, 4 were hospitalized between 5 and 10 days. Twenty participants (95%) reported having been vaccinated before being infected with COVID-19, while one did not provide an answer. No statistical difference between the groups was found on any characteristics. Randomization was intended to promote comparability between groups; observed numerical differences (e.g., sex and diabetes) were small, and effect sizes were reported for descriptive purposes only and were not considered clinically meaningful.

**Table 2:** Demographics of the study groups

	In-person group (*N* = 10)	Telerehabilitation group (*N* = 11)	Effect size
Sociodemographic data			
Age (years)	51.7 ± 17.6	54.2 ± 11.0	− 0.2 (95% CI − 1.0–0.7)
M: F	6: 4	3: 8	0.3 (95% CI − 0.1–0.7)
Height (m)	1.7 ± 0.1	1.7 ± 0.1	0.3 (95% CI − 0.6–1.1)
Weight (kg)	87.0 ± 16.7	82.0 ± 23.3	0.2 (95% CI − 0.6–1.1)
BMI (kg/m^2^)	31.0 ± 6.5	29.6 ± 7.0	0.2 (95% CI − 0.7–1.1)
Working status before rehabilitation			
Medical sick leave, *n* (%) Working, *n *(%) Non-applicable, *n* (%)	7 (70%) 1 (10%) 2 (20%)	9 (80%) 1 (10%) 1 (10%)	0.2 (95% CI − 0.3–0.6)
Baseline characteristics			
Smoking			
Yes, *n* (%) No, *n* (%)	1 (10%) 9 (90%)	1 (9%) 10 (91%)	− 0.0 (95% CI − 0.2–0.3)
Heart rate (bpm)	92 ± 12	84 ± 10	0.7 (95% CI − 0.2–1.6)
SpO₂ (%)	97 ± 2	97 ± 2	− 0.3 (95% CI − 1.1–0.6)
Systolic blood pressure (mmHg)	132 ± 20	137 ± 22	− 0.2 (95% CI − 1.1–0.6)
Diastolic blood pressure (mmHg)	77 ± 11	85 ± 15	− 0.6 (95% CI − 1.5–0.3)
Comorbidities			
High blood pressure (> 140/90 mmHg)			
Yes, *n* (%) No, *n* (%)	2 (20%) 8 (80%)	4 (36%) 7 (64%)	0.2 (95% CI (− 0.2–0.5)
Diabetes mellitus (fasting blood glucose ≥ 6.11 mmol/L)			
Yes, *n* (%) No, *n* (%)	0 (0%) 10 (100%)	2 (18%) 9 (82%)	0.3 (95% CI − 0.0–0.4)
Asthma			
Yes, *n* (%) No, *n* (%)	4 (40%) 6 (60%)	3 (27%) 8 (73%)	− 0.1 (95% CI − 0.3–0.5)
COPD			
Yes, *n* (%) No, *n* (%)	1 (10%) 9 (90%)	0 (0%) 11 (100%)	− 0.2 (95% CI − 0.1–0.3)
ASCVD			
Yes, *n* (%) No, *n* (%)	2 (20%) 8 (80%)	1 (9%) 10 (91%)	− 0.2 (95% CI −.0.2–0,4)
Respiratory function			
FEV_1_ (L)	3.1 ± 0.8	2.7 ± 0.7	0.5 (95% CI − 0.3–1.3)
FEV_1_ (%)	96.6 ± 16.3	90.2 ± 14.9	0.4 (95% CI − 0.5–1.3)
TLC (L)	5.8 ± 1.1	5.7 ± 1.0	0.1 (95% CI − 0.7–1.0)
TLC (%)	97.8 ± 17.4	97.3 ± 14.0	0.0 (95% CI − 0.8–0.9)
FVC (L)	3.8 ± 1.1	3.5 ± 0.9	0.3 (95% CI − 0.5–1.2)
FVC (%)	96.0 ± 16.1	97.5 ± 15.2	− 0.1 (95% CI − 1.0–0.8)
DLCO (%)	94.9 ± 30.9	96.4 ± 12.4	− 0.1 (95% CI − 0.9–0.8)

± indicates standard deviation, *M* male, *F* female,* BMI* Body Mass Index, *SpO₂* Oxygen saturation, *COPD* chronic obstructive pulmonary disease, *ASCVD* atherosclerotic cardiovascular disease, *CFS* Clinical Frailty Scale, *SPPB* Short Physical Performance Battery, *FEV*_*1*_ forced expired volume in one second, *FVC* forced vital capacity, *TLC* total lung capacity, *DLCO *diffusing capacity of the lungs for carbon monoxide

### Primary outcome measures

#### Feasibility

##### Recruitment and attrition rate

**Fig. 1:**
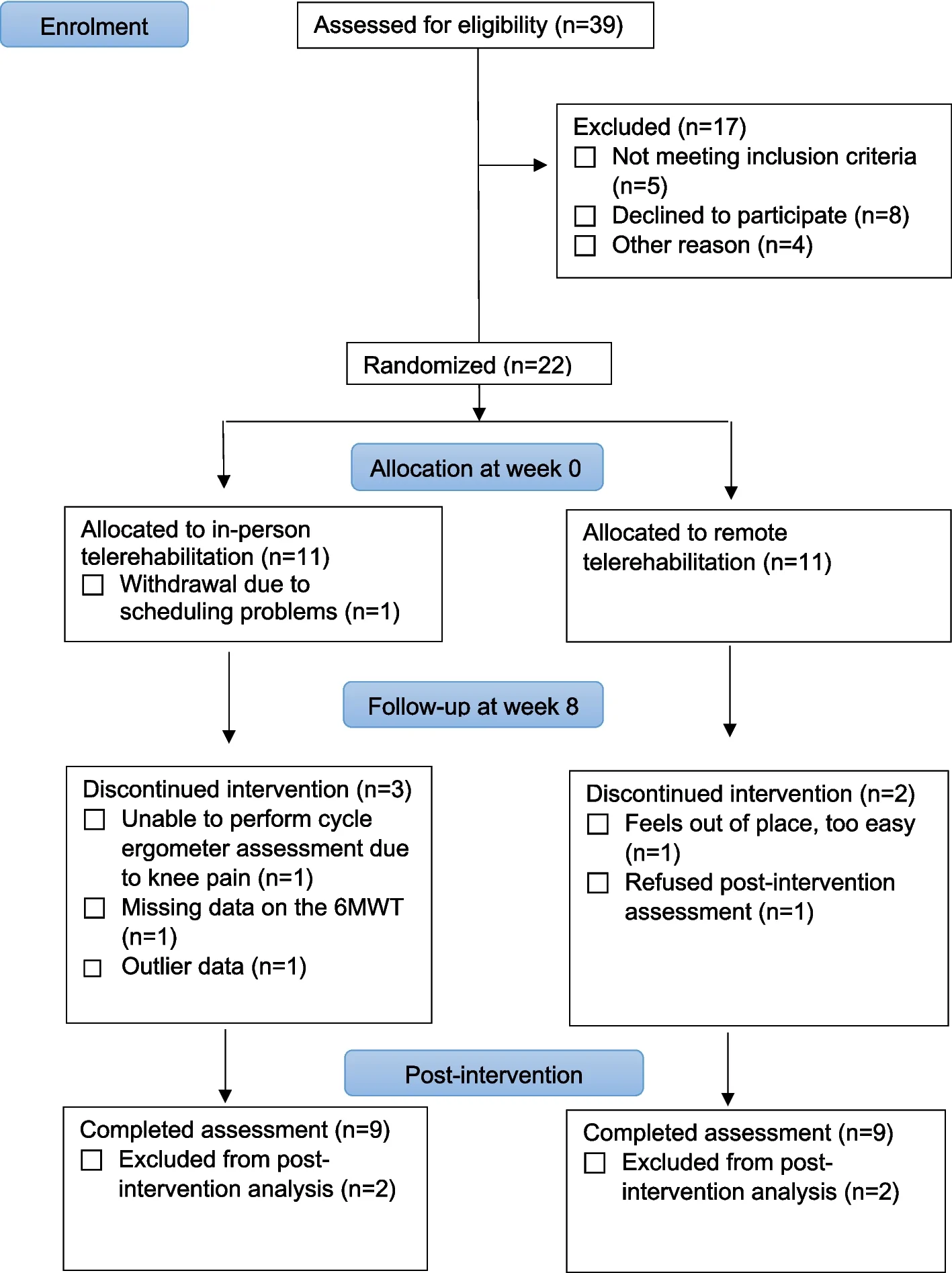
CONSORT flowchart for reporting RCT

Over 7 months, 39 eligible individuals were approached, and 22 consented to participate in the study (56.4% consent rate) (Fig. 1). The main reason for declining participation was transportation issues (5 out of the 8 individuals who declined, among the 17 excluded). Randomization was successfully completed for all 22 consented participants. One participant in the in-person group withdrew before starting the program due to scheduling conflicts. All 21 participants who initiated the program completed the baseline assessment. Among the 11 participants in the remote rehabilitation group, 1 (9%) withdrew because the program was not sufficiently demanding for their physical condition, and another completed the intervention but did not attend the final assessments for unspecified reasons. In the in-person group, one participant could not perform the cardiovascular exercise test due to knee pain unrelated to the program but completed other assessments. Two additional cases involved missing or implausible post-intervention data (1 outlier of 81 repetitions on the 1STST and one missing 6MWT value). After excluding these cases, 9 participants per group (85.7%) were included in the post-intervention analyses, resulting in an attrition rate of 14.3%.

##### Adherence to the rehabilitation program

The participants included in the in-person group performed the rehabilitation program an average of 2.4 days per week, and for their part, the participants in the telerehabilitation group did it 2.7 days per week. Three participants in each group have completed all the sessions included in the protocol. Eight participants in the in-person group and ten participants in the telerehabilitation group attended at least 75% of the sessions. Two participants (20%) in the in-person group attended less than 50% of the sessions, and one participant in the telerehabilitation group dropped out, as stated earlier. Reasons for non-adherence to the rehabilitation program included health problems (e.g., bronchitis, migraine) unrelated to the program, as well as personal issues and familial issues.

##### Adverse events and satisfaction with rehabilitation

No serious adverse events related to the intervention were recorded. The symptoms most frequently reported by participants (i.e., fatigue, muscle pain, dyspnea, confusion, headaches) were consistent with Long COVID and did not meet the study’s predefined criteria for adverse events, as defined in the “Methods” section.

Participant’s satisfaction levels were high, with 100% finding the quality of services to be excellent (80%) or good (20%) in the in-person group and 90% strongly agreeing with the quality of services received in the telerehabilitation group. Similarly, 100% of participants would recommend the rehabilitation program they have received, whether in-person or virtual, and would use the rehabilitation services again if necessary. For the telerehabilitation group, the question that received the lowest score (4.2/5) was Item 5 (“I do not need assistance while using the system”; Table 4), suggesting that some participants perceived a need for assistance when using the platform. Tables 3 and 4 show post-rehabilitation scores on participant CSQ-8 and on the Telemedicine Patient Satisfaction Questionnaire.

**Table 3:** Post-rehabilitation scores on the participant satisfaction questionnaire 8 (CSQ-8)

	Scale score				
Item	1	2	3	4	Average (SD)
1. How would you rate the quality of service you have received?	0	0	20%	80%	3.8(0.4)
2. Did you get the kind of service you wanted?	0	0	60%	40%	3.4 (0.5)
3. To what extent did the program meet your needs?	0	10%	40%	50%	3.4 (0.7)
4. If a friend were in need of similar help, would you recommend the program to him or her?	0	0	40%	60%	3.6 (0.5)
5. How satisfied are you with the amount of help you have received?	0	0	50%	50%	3.5 (0.5)
6. Have the services you received helped you deal with your problems more effectively?	0	10%	10%	80%	3.7 (0.7)
7. In an overall general sense, how satisfied are you with the services you have received?	0	0	30%	70%	3.7 (0.5)
8. If you were to seek help again, would you come back to the service?	0	0	50%	50%	3.5 (0.5)

**Table 4:** Post-telerehabilitation scores on the participant Telemedicine Patient Satisfaction Questionnaire

	Scale score					
Item	Totally disagree 1	Disagree 2	Neutral 3	Agree 4	Totally agree 5	Average (SD)
1. I can easily talk to my health-care provider	0	0	10%	20%	70%	4.6 (0.7)
2. I can hear my health-care provider clearly	0	0	0	20%	80%	4.8 (0.4)
3. My health-care provider is able to understand my health-care condition	0	0	0	20%	80%	4.8 (0.4)
4. I can see my health-care provider as if we met in-person	0	0	0	40%	60%	4.6 (0.5)
5. I do not need assistance while using the system	0	0	20%	40%	40%	4.2 (0.8)
6. I feel comfortable communicating with my health-care provider	0	0	0	20%	80%	4.8 (0.4)
7. I think the health-care provided via telemedicine is consistent	0	0	0	20%	80%	4.8 (0.4)
8. I obtain better access to health-care services by use of telemedicine	0	0	0	20%	80%	4.8 (0.4)
9. Telemedicine saves me time travelling to hospital or a specialist clinic	0	0	0	0	100%	5(0)
10. I do receive adequate attention	0	0	0	20%	80%	4.8 (0.4)
11. Telemedicine provides for my health-care need	0	0	0	50%	50%	4.5 (0.5)
12. I find telemedicine an acceptable way to receive health-care services	0	0	0	20%	80%	4.8 (04)
13. I will use telemedicine services again	0	0	0	20%	80%	4.8 (0.4)
14. Overall, I am satisfied with the quality of service being provided via telemedicine	0	0	0	10%	90%	4.9 (0.3)
15. I would recommend telerehabilitation services to a friend or family member	0	0	0	10%	90%	4.9 (0.3)

### Secondary outcomes measures

#### Preliminary estimate of effect of the telerehabilitation program compared to the in-person program on pedaling time (exercise capacity)

**Fig. 2:**
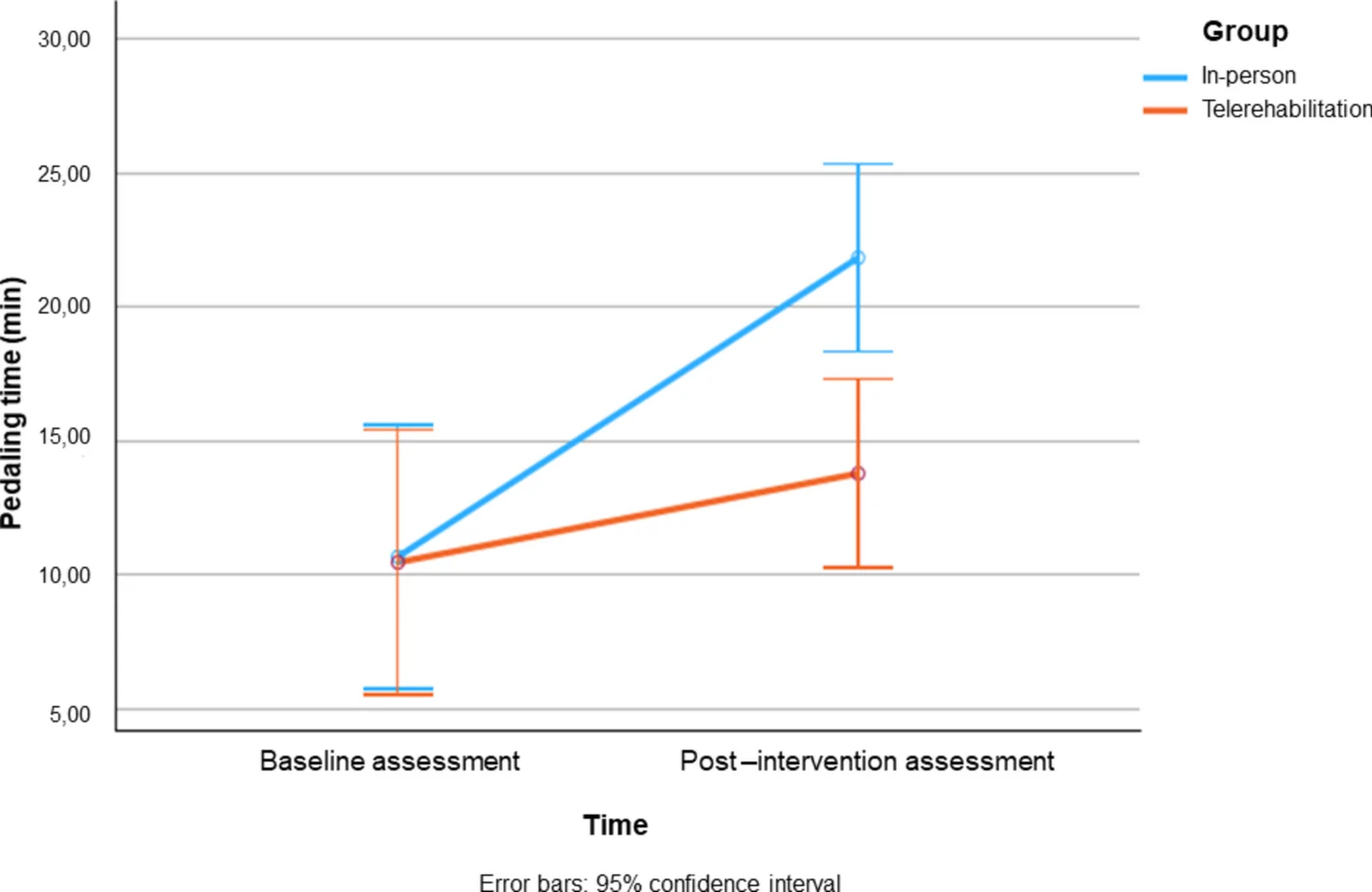
Between-group comparison over time for pedaling time (exercise capacity)

Results from the between-group comparison over time for the exercise capacity are presented in Fig. 2. There was a group-by-time interaction (*F*_(1,16)_ = 6.1, ηp^2^ = 0.28), indicating there was a difference between groups over time, with participants in the in-person group having a greater improvement in their pedaling time. There was a effect of time (*F*_(1,16)_ = 20.9, ηp^2^ = 0.57, 95% CI = − 10.6–− 3.9), indicating that the pedaling time improved after the intervention in both groups. However, there was no main effect of group (*F*_(1,16)_ = 3.0, ηp^2^ = 0.16, 95% CI = − 0.9–9.2).

#### Functional ability and independence, clinical frailty, and quality of life

Between-group comparisons over time for the 6MWT and the 1STST are presented in Table 5. Baseline scores for the Barthel Index, CFS, and SPPB are presented in Table 6. There was only a main effect of time for the 1STST (*F*_(1,16)_ = 8.1, ηp^2^ = 0.34, 95% CI − 9.0–− 1.3), indicating that both groups improved lower limb strength and endurance.

**Table 5:** Between-group comparison for 6MWT and 1STST

Variable	In-person group (*N* = 9) (Mean ± SD)	Telerehabilitation group (*N* = 9) (Mean ± SD)	Main effect of group ηp^2^	Main effect of time ηp^2^	Group*time interaction ηp^2^
6MWT (m)					
Baseline	408.0 ± 90.0	445.1 ± 102.1	0.0 (95% CI − 98.1–66.8)	0.1 (95% CI − 44.0–14.5)	0.1
Post-intervention	444.2 ± 78.9	438.4 ± 77.0			
1STST					
Baseline	20.4 ± 9.7	19.4 ± 6.1	0.1 (95% CI − 2.8–10.9)	0.3 (95% CI − 9.0–− 1.3)	0.2
Post-intervention	28.7 ± 9.1	21.6 ± 5.8			

*6MWT* = 6 Minute Walking Time, *1STST* = 1 min sit to stand, *SD* = standard deviation

**Table 6:** Between-group comparison for functional capacity and independence

Variable	In-person group (*N* = 10)	Telerehabilitation group (*N* = 11)	Mann–Whitney result
Functional capacity and independence			
Barthel Index	100.0 ± 0.0	96.8 ± 4.6	*U* = 35, *Z* = − 2.1, *r* = 0.5
CFS	3.8 ± 0.4	4.2 ± 0.9	*U* = 71, *Z* = 1.2, *r* = 0.3
SPPB	10.6 ± 1.5	10.2 ± 1.9	*U* = 50, *Z* = − 0.4, *r* = 0.1

*U* Mann–Whitney *U* statistic, *Z* standardized test statistic, *r* effect size, *CFS* Clinical Frailty Scale, *SPPB* Short Physical Performance Battery

Table 6 presents baseline scores for the Barthel Index (0–100), the CFS (1–9), and SPPB (0–12). No statistical differences between groups were found at baseline, and scores remained stable post-intervention, indicating participants were generally independent (Barthel near maximum), not frail (CFS in the lower range), and had good physical function (SPPB > 9).

Quality of life scores from the SGRQ could not be analyzed due to missing baseline data; however, self-rated health improved: before the program, 52.6% reported “average” health, while after rehabilitation, 36.8% reported “good” health and 26.3% “average” health (Table 7).

**Table 7:** Pre- and post-scores on participant perspective of their current state of health

Your current state of health is:	Very good (0)	Good (1)	Average (2)	Bad (3)	Very bad (4)
In-person					
Pre	0	30%	50%	20%	0
Post	0	50%	40%	10%	0
Telerehabilitation					
Pre	0	11.1%	60%	33.3%	0
Post	0	22.2%	33.3%	44.4%	0

#### Sample size estimation for the definitive trial

Based on the pedaling time outcome (cycle ergometer endurance test), which showed a Group x Time interaction favoring the in-person group, and considering a large effect size (Cohen’s *f* = 0.61 derived from ηp^2^), an alpha level of 0.05, a power of 90%, and accounting for a 15% attrition rate, the sample size estimation for a full RCT indicated that 36 participants would be needed (18 per group). However, this estimate should be interpreted with caution, as it is based on the effect size observed in this small pilot study, which may be subject to considerable variability and overestimation.

## Discussion

This pilot randomized controlled trial demonstrates that this study was feasible and appeared safe at the clinical site in this current form for the population included in this study, as no adverse events were reported. Participants had access to the research team and kinesiologist at any point during the study period and did not report any adverse symptoms. These findings support the suitability of supervised, group-based telerehabilitation as a delivery mode in this context. This is in line with the literature that has demonstrated the feasibility and safety of group-based supervised telerehabilitation for patients with Long COVID [50–52].

As part of this study, the feasibility of group-based telerehabilitation for Long COVID was assessed using recruitment rate, dropout rate, adherence rate to the rehabilitation program, occurrence of adverse events, and satisfaction. The recruitment nurse was able to obtain a consent rate of 56% among invited patients. The main reason not to take part in the study was the transportation in cases where the participants were randomly placed in the in-person group. It should be noted that Trois-Rivières hospital covers a vast territory, which may explain why some patients from neighboring towns did not want to make the three-times-a-week round trip to the kinesiology clinic. This finding underscores the potential benefit of integrating telerehabilitation into the Trois-Rivières hospital’s service offering to improve access and reduce travel-related barriers for patients living in remote areas.

As previously stated, a sample of 36 patients (18 per group) would be required to conduct an RCT with enough power to find significant changes between the groups for the exercise capacity outcome. This estimate was based on a large effect size (> 0.6) and should be interpreted cautiously, as recent literature suggests smaller effects in similar populations [53]. Given the current consent rate, achieving the necessary number of participants would likely be feasible by increasing the available time slots at the kinesiology clinic and enhancing recruitment efforts, such as leveraging digital advertising. Adherence to the rehabilitation program was high for both modes of delivery, and dropouts were low, suggesting that rehabilitation in either way was well-received by the participants. Only one participant dropped out from the telerehabilitation group because it was too easy for them. There was probably a mismatch between user needs and intervention components.

To address this in future implementations, it would be important to systematically assess each participant’s expectations and needs before starting rehabilitation. This step would allow tailoring modified exercises to individual requirements while maintaining the group-based teaching approach led by the kinesiologist. Such personalization could improve engagement and effectiveness of the intervention [54].

No serious adverse events related to the intervention were recorded during the course of this study, regardless of whether participants received in-person or virtual sessions. This finding supports the safety of both delivery modes for participants meeting the study’s inclusion criteria and are consistent with previous reports on the safety of telerehabilitation in Long COVID population [18, 50, 51].

Participants’ satisfaction was also high and similar in both groups, proving the acceptability of the intervention and telerehabilitation. It is important to know how satisfied participants are with the telerehabilitation because it influences compliance and motivation towards intervention [55]. However, given the score concerning the need for system support (i.e., videoconference) on the telerehabilitation questionnaire and to make sure every patient has the knowledge of and the ability to engage in telerehabilitation, it would be relevant to offer training on telecommunication applications before the start of the intervention [56].

Preliminary results related to effects showed an improvement in exercise capacity over time for both groups, although greater in the in-person group. There was also an improvement in the number of repetitions during 1STST at follow-up for both groups, but it remains low with around 83% of the participants being under the 2.5th percentile value [57]. No effect was found for functional independence, degree of frailty, or functional mobility. At baseline, participants’ scores on the Barthel Index, CFS, and SPPB indicated functional independence, low frailty, and preserved physical performance. These results suggest that participants did not present serious functional limitations, which made further improvement more difficult to demonstrate. Also, the cohort consisted mainly of younger participants without comorbidities who did not require hospital admission for COVID-19, which may explain the favorable baseline results. While individuals who required hospitalization during acute illness are more likely to present functional limitations and ongoing rehabilitation needs, those with milder initial disease may still experience persistent symptoms [24]. This highlights the importance of assessing each patient’s capacity to adapt the rehabilitation program accordingly. This study was not designed as an equivalence trial; comparisons between groups are exploratory and intended to inform future research. Both the in-person and telerehabilitation programs were developed specifically for this study based on the same rehabilitation principles, with adaptations for remote delivery in the telerehabilitation group.

Regarding quality of life, considering the high amount of missing data at baseline, a validated tool such as the European Quality-of-Life 5 Dimensions Questionnaire (EQ-5D) could be considered in future studies, as it provides a concise and generic measure of self-reported health. However, although EQ-5D is widely used in Long COVID research, its ability to capture fluctuating symptoms remains limited, so further validation and complementary measures may be needed [58].

For the 6MWT, the results showed no effect of the rehabilitation program as opposed to other studies in the field. However, from a clinical point of view, it has been suggested that a > 30.5 m increase for 6MWT is a minimal clinically important difference for patients with cardiopulmonary diseases [59]. In that case, the in-person group demonstrated a 36.22-m increase, whereas the telerehabilitation group did not show clinical improvement. Similar to the cycle ergometer endurance test, in-person groups appear to be making more significant progress. Considering that all participants received the same rehabilitation program, the adaptation of exercises by the kinesiologist was perhaps more targeted to the in-person group. The kinesiologist may have been better able to assess the level of effort given by each in-person participant. For in-person participants, it may also have been more reassuring to have direct support from the professional. Concerns about symptom exacerbation have been reported among individuals with Long COVID [60]. During the final assessments, some participants chose to stop testing early due to concerns about worsening their symptoms, which may have influenced performance. Given the small sample size, these observations are exploratory and intended to inform future research rather than provide definitive conclusions.

## Limitations

This study has several limitations to consider. First, the sample was small and recruited by convenience, and the study was conducted in a single facility in Trois-Rivières, Québec, which limits generalizability. In addition, 14% of participants were unable to complete all follow-up measurements, potentially introducing bias. All participants in the telerehabilitation group were required to have internet access and appropriate computer equipment, which may further restrict applicability to the general population. Participants were not blinded to group allocation due to the nature of the intervention, which could influence preliminary effect outcomes.

The rehabilitation program used was based on 2021 guidelines [24] and did not incorporate adaptations for considerations that have been increasingly recognized in the context of Long COVID, including those relevant to post-infectious symptom responses. Current recommendations mention these issues without providing clear definitions or guidance for their management, which represents an important gap in practice and should be addressed in future updates. Although exercise intensity was monitored using the RPE scale, this measure reflects effort during the session and does not capture delayed onset of symptoms that may occur hours or days later (e.g., post-exertional symptom exacerbation). Symptom status was not systematically assessed after performance-based outcome measures, which limits the ability to fully capture potential delayed responses to exercise. Participants were not assessed for post-exertional malaise (PEM) at baseline (i.e., delayed worsening of symptoms following exertion); therefore, we cannot determine whether PEM was present as part of their Long COVID phenotype. Consequently, the findings may not be generalizable to individuals with Long COVID who experience prominent post-exertional symptom exacerbation/PEM. Finally, given the small sample size and the number of clinical outcomes assessed, these preliminary findings are exploratory and intended to inform future research rather than provide definitive conclusions. Feasibility outcomes were interpreted descriptively without predefined thresholds, which is consistent with the pilot nature of this study; however, the absence of explicit progression criteria to guide the decision to proceed to a definitive trial represents a limitation. Future studies should establish specific feasibility criteria and clearly defined progression criteria to support interpretation and planning. The trial was registered after the first participant enrollment, which represents a limitation and should be avoided in future studies.

## Conclusion

In conclusion, this pilot randomized controlled study demonstrated the feasibility and safety of delivering a supervised group-based rehabilitation intervention remotely to individuals with Long COVID characterized by persistent dyspnea 8 weeks post-infection. The protocol was feasible in the clinic with respect to recruitment rate, dropout rate, adherence, occurrence of adverse events, and satisfaction. These findings apply to a relatively young, non-hospitalized population and should not be generalized to all Long COVID phenotypes, particularly those characterized by marked exercise intolerance. Preliminary observations on the effects are exploratory and intended to inform future research rather than provide definitive conclusions. Further studies with larger, multicenter samples and careful phenotyping are needed to confirm these effects and explore generalizability.

## Data Availability

The data that support the findings of this study are available from the corresponding author upon reasonable request.

## Abbreviations

1STST: One-Minute Sit-to-Stand Test
6MWT: 6-Minute Walk Test
ADL: Activities of daily living
ASCVD: Atherosclerotic cardiovascular disease
BMI: Body Mass Index
CDC: Centers for Disease Control and Prevention
CERT: Consensus in Exercise Reporting Template
CFS: Clinical Frailty Scale
CONSORT: Consolidated Standards of Reporting Trials
COPD: Chronic Obstructive Pulmonary Disease
CSQ-8: Customer Satisfaction Questionnaire
DLCO: Diffusing capacity of the lungs for carbon monoxide
EQ-5D: European Quality-of-Life 5 Dimensions Questionnaire
FEV_1_: Forced expired volume in one second
FVC: Forced vital capacity
INESSS: Institut national d’excellence en santé et en services sociaux
IRB: Institutional Review Board
IUCPQ: Institut Universitaire de Cardiologie et de Pneumologie de Québec
mMRC: Modified Medical Research Council dyspnea scale
PCR: Positive polymerase chain reaction
PEM: Post-exertional malaise
RCT: Randomized controlled trial
RPE: Rate of perceived exertion
SARS-CoV-2: Severe Acute Respiratory Syndrome Coronavirus 2
SGRQ: St. George’s Respiratory Questionnaire
SPPB: Short Physical Performance Battery test
SPSS: Statistical Package for the Social Sciences
TLC: Total lung capacity
UETMIS: Unité d’évaluation des technologies et des modes d’intervention en santé
UQTR: Université du Québec à Trois-Rivières
VO_2_max: Maximal oxygen uptake
